# A retrospective analysis of *RET* translocation, gene copy number gain and expression in NSCLC patients treated with vandetanib in four randomized Phase III studies

**DOI:** 10.1186/s12885-015-1146-8

**Published:** 2015-03-23

**Authors:** Adam Platt, John Morten, Qunsheng Ji, Paul Elvin, Chris Womack, Xinying Su, Emma Donald, Neil Gray, Jessica Read, Graham Bigley, Laura Blockley, Carl Cresswell, Angela Dale, Amanda Davies, Tianwei Zhang, Shuqiong Fan, Haihua Fu, Amanda Gladwin, Grace Harrod, James Stevens, Victoria Williams, Qingqing Ye, Li Zheng, Richard de Boer, Roy S Herbst, Jin-Soo Lee, James Vasselli

**Affiliations:** 1 AstraZeneca, da Vinci Building, Melbourn Science Park, Cambridge Road, Melbourn, Royston, Hertfordshire SG8 6HB UK; 2 AstraZeneca, Macclesfield, UK; 3 Innovation Cancer Center, AstraZeneca R&D, Shanghai, China; 4 Department of Hematology & Medical Oncology, Western Hospital, Melbourne, Victoria Australia; 5 Yale Comprehensive Cancer Center, New Haven, CT USA; 6 National Cancer Center, Goyang, Republic of Korea; 7 AstraZeneca, Wilmington, DE USA; 8 Current address – MedImmune, Gaithersburg, MD USA

**Keywords:** *RET* rearrangement, Vandetanib, Non-small-cell lung cancer

## Abstract

**Background:**

To determine the prevalence of *RET* rearrangement genes, *RET* copy number gains and expression in tumor samples from four Phase III non-small-cell lung cancer (NSCLC) trials of vandetanib, a selective inhibitor of VEGFR, RET and EGFR signaling, and to determine any association with outcome to vandetanib treatment.

**Methods:**

Archival tumor samples from the ZODIAC (NCT00312377, vandetanib ± docetaxel), ZEAL (NCT00418886, vandetanib ± pemetrexed), ZEPHYR (NCT00404924, vandetanib vs placebo) and ZEST (NCT00364351, vandetanib vs erlotinib) studies were evaluated by fluorescence *in situ* hybridization (FISH) and immunohistochemistry (IHC) in 944 and 1102 patients.

**Results:**

The prevalence of *RET* rearrangements by FISH was 0.7% (95% CI 0.3–1.5%) among patients with a known result. Seven tumor samples were positive for *RET* rearrangements (vandetanib, *n* = 3; comparator, *n* = 4). 2.8% (*n* = 26) of samples had *RET* amplification (innumerable *RET* clusters, or ≥7 copies in > 10% of tumor cells), 8.1% (*n* = 76) had low *RET* gene copy number gain (4–6 copies in ≥40% of tumor cells) and 8.3% (*n* = 92) were RET expression positive (signal intensity ++ or +++ in >10% of tumor cells). Of *RET*-rearrangement-positive patients, none had an objective response in the vandetanib arm and one patient responded in the comparator arm. Radiologic evidence of tumor shrinkage was observed in two patients treated with vandetanib and one treated with comparator drug. The objective response rate was similar in the vandetanib and comparator arms for patients positive for *RET* copy number gains or RET protein expression.

**Conclusions:**

We have identified prevalence for three RET biomarkers in a population predominated by non-Asians and smokers. *RET* rearrangement prevalence was lower than previously reported. We found no evidence of a differential benefit for efficacy by IHC and *RET* gene copy number gains. The low prevalence of *RET* rearrangements (0.7%) prevents firm conclusions regarding association of vandetanib treatment with efficacy in the *RET* rearrangement NSCLC subpopulation.

**Trial registration:**

Randomized Phase III clinical trials (NCT00312377, ZODIAC; NCT00418886, ZEAL; NCT00364351, ZEST; NCT00404924, ZEPHYR).

**Electronic supplementary material:**

The online version of this article (doi:10.1186/s12885-015-1146-8) contains supplementary material, which is available to authorized users.

## Background

Cancer treatment paradigms are evolving to exploit the sensitivity of tumors to inhibitors that target the products of genes carrying driver mutations [1]. A number of genetic aberrations that drive and maintain tumorigenesis have recently been identified in non-small-cell lung cancer (NSCLC). These include fusion genes generated by chromosomal rearrangements between the rearranged during transfection (*RET*) gene and other genes, most commonly kinesin family 5B (*KIF5B*) and coiled coil domain containing-6 (*CCDC6*) [2-12]. These fusions lead to overexpression of truncated RET proteins containing the RET kinase domain, which can induce transformation and occur in tumors that rarely harbor mutations in other common drivers, ie epidermal growth factor receptor (*EGFR*), *KRAS*, human epidermal growth factor receptor, and anaplastic lymphoma receptor (*ALK*) genes. *RET* rearrangement was first shown to be associated with papillary thyroid carcinoma (PTC), leading to the fusion oncoprotein (*RET/PTC*) and constitutive activation of RET receptor tyrosine kinase in papillary cancer cells [13]. In addition, *RET* mutations are present in the germline of nearly all patients with hereditary forms of medullary thyroid cancer (MTC) [14-16] and approximately 50% of patients with sporadic MTC have somatic *RET* mutations that are associated with a worse outcome [17].

Vandetanib is a once-daily, oral anticancer agent that selectively inhibits vascular endothelial growth factor receptor (VEGFR), RET and EGFR signaling [18,19], and is licensed for the treatment of MTC in several geographical regions. Preclinical studies have demonstrated that vandetanib inhibits RET signaling arising from *RET* mutations in a MTC cell line and inhibits growth of human PTC cell lines that carry spontaneous *RET/PTC* rearrangements [19]. In addition, vandetanib has been shown to inhibit the proliferation of cells expressing *RET-KIF5B* [2,3] and a human lung adenocarcinoma cell line harboring an endogenous *RET-CCDC6* [20].

Four randomized Phase III clinical trials have evaluated the efficacy of vandetanib in NSCLC in combination with docetaxel (NCT00312377; ZODIAC [21]), in combination with pemetrexed (NCT00418886; ZEAL [22]) or as a monotherapy (NCT00364351; ZEST [23] and NCT00404924; ZEPHYR [24]). These studies in unselected patient populations demonstrated antitumor activity of vandetanib, but there was no overall survival benefit when added to standard chemotherapy or as monotherapy versus erlotinib [21-24]. The aim of this study, a retrospective evaluation of tumor samples from the NSCLC Phase III program, was to determine the prevalence of *RET* rearrangements and other potential RET biomarkers within this population and to investigate any association with outcome to vandetanib treatment.

## Methods

### Overview of NSCLC studies

#### Study treatments and assessment of efficacy

All four studies are registered at www.clinicaltrials.gov and were Phase III, multicenter, randomized, double-blind studies conducted in 25 (ZODIAC), 21 (ZEAL), 22 (ZEPHYR) and 22 (ZEST) countries, respectively. Enrollment was conducted between 2006 and 2008. Full details of the study design and methodology have been reported previously [21-24]. Briefly, in ZODIAC (NCT00312377), patients received docetaxel in combination with oral vandetanib 100 mg/day (*n* = 694) or matched placebo (*n* = 697). Docetaxel 75 mg/m^2^ was administered as an intravenous infusion every 21 days, for a maximum of six cycles. In ZEAL (NCT00418886), patients received pemetrexed in combination with oral vandetanib, 100 mg/day (*n* = 256) or matched placebo (*n* = 278). Pemetrexed 500 mg/m^2^ was administered as an intravenous infusion every 21 days, for a maximum of six cycles. Vandetanib was evaluated as a monotherapy in two of the studies: in ZEPHYR (NCT00404924), patients received vandetanib 300 mg/day (*n* = 617) or placebo (*n* = 307); and in ZEST (NCT00364351), patients received vandetanib 300 mg/day (*n* = 623) or erlotinib 150 mg/day (*n* = 617). Objective tumor assessments were categorized using Response Evaluation Criteria in Solid Tumors (RECIST; version 1.0).

#### Patient eligibility

Data were evaluated from adult patients with histologically or cytologically confirmed locally advanced or metastatic (stage IIIB to IV) NSCLC after failure of: first-line anticancer therapy (ZODIAC and ZEAL); one or two chemotherapy regimens (ZEST); or an EGFR inhibitor following one or two chemotherapy regimens (ZEPHYR). For all studies, inclusion criteria included: World Health Organization performance status 0–2; life expectancy ≥12 weeks and no significant hematologic, hepatic, renal or cardiac abnormalities. Patients with squamous cell histology were also eligible. Prior treatment with VEGFR inhibitors (all studies), docetaxel (ZODIAC only), pemetrexed (ZEAL only) and EGFR inhibitors (ZEST only) was not permitted. Prior treatment with bevacizumab (all studies) and cetuximab (ZEST only) was allowed.

#### Ethics statements

All patients provided written informed consent, the trials were approved by all relevant institutional ethical committees or review bodies (The University of Texas MD Anderson Cancer Center Surveillance Committee, Houston, TX, USA; The Royal Melbourne Research Foundation, Melbourne, Australia; Institutional Review Board of National Cancer Center, Gyeonggi-do, Republic of Korea; Cedars-Sinai Medical Center Institutional Review Board, Beverly Hills, CA, USA) and was conducted in accordance with the Declaration of Helsinki, Good Clinical Practice, and the AstraZeneca policy on Bioethics [25]. Data were generated in accordance with the Medical and Healthcare Products Regulatory Agency Good Clinical Practice Guidelines for laboratories [26].

#### Samples

Archival tumor specimens were sampled prior to enrollment of patients onto study. Provision of these samples for genetic/immunohistochemical (IHC) assessment was not compulsory in any study, resulting in collection from approximately one third of patients. There was no observable bias to sampling consent. Tumor samples were supplied as formalin-fixed paraffin-embedded biopsies, resections or sections and could be obtained at any time during the respective study. Cell lines used as controls in IHC were supplied as cell pellets and stored at −80°C prior to use. All analyses were carried out in AstraZeneca laboratories in the UK and China.

### Assay methods

#### Fluorescence in situ hybridization (FISH) analysis

**Figure 1:**
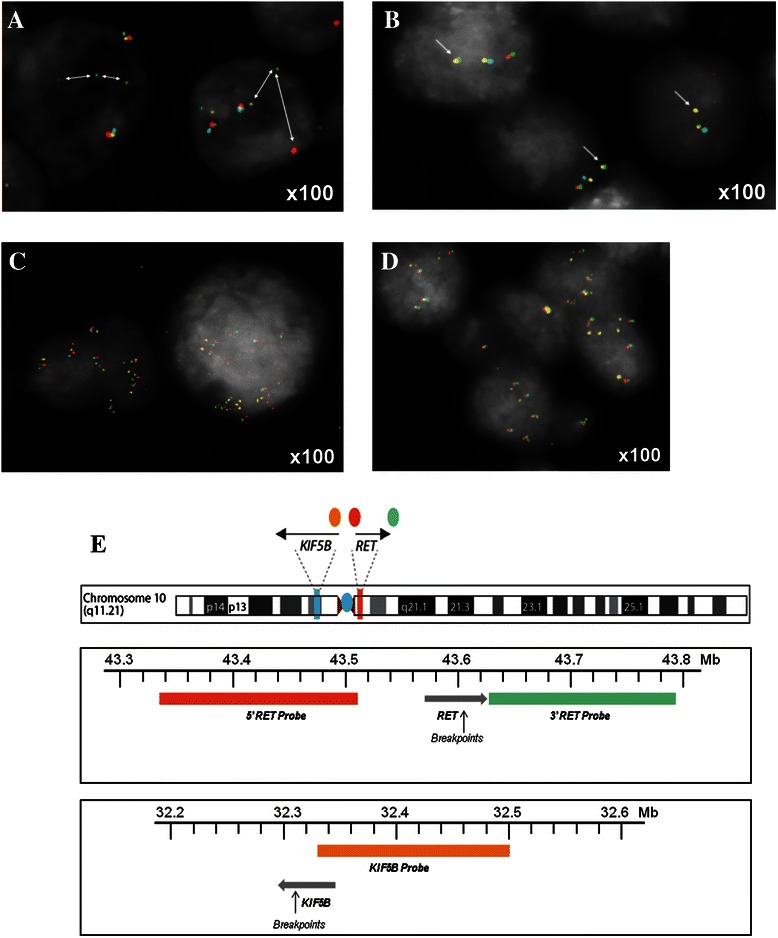
**Representative FISH images. (A)** Unknown *RET* rearrangement, **(B)***RET-KIF5B* fusion, **(C)***RET* gene amplifications and **(D)** low *RET* gene copy number gain. **(E)** Loci for RET FISH probes.

*RET-KIF5B*, alternative *RET* rearrangements and *RET* gene amplifications were identified using a FISH probe set of four fluorescent-labeled bacterial artificial chromosome (BAC) clone-derived DNA probes designed in-house: RP11-124O11 (located upstream of *RET*) labeled with spectrum red; RP11-718J13 (located immediately downstream of *RET*) labeled with spectrum green (fluorescein isothiocyanate); RP11-983E11 (located upstream of *KIF5B* exon 2) labeled with spectrum gold (5[6]-carboxyrhodamine 6G deoxyuridine-5′-triphosphate [dUTP]); and centromere of chromosome 10 labeled with spectrum aqua (diethylaminocoumarin-5-dUTP; Figure 1). For tissue samples assessed at the UK site, these probes were sourced from Empire Genomics LLC, Buffalo, NY; for tissue samples assessed at the China site, the probes were generated in-house. BACs and labels used at both sites were identical. Sections were processed with reagents from the Histology FISH accessory kit (Dako, Dako Corporation, Carpinteria, CA, USA, cat #K5799) according to the manufacturer’s instructions. Briefly at the UK site, formalin-fixed, paraffin-embedded sections (4 μm) were mounted on glass slides. Sections were deparaffinized in xylene, hydrated through a graded ethanol series, and then incubated with the accessory kit pretreatment solution at 95–100°C for 10 minutes. The sections were then washed and pepsin digestion was carried out at 37°C in a Dako hybridizer for 5 minutes; the sections were dehydrated through ethanol and allowed to air dry. The fluorescent probe mix was applied and then the probe and section co-denatured in a Dako hybridizer at 83°C for 3 minutes followed by overnight incubation at 37°C. The sections were washed in 1x saline-sodium citrate (SSC)/0.3% Igepal at 72°C for 2 minutes, followed by 2x SSC at room temperature, before dehydration through ethanol. Sections were mounted in Dako antifade mounting media (Dako, #K5799). The procedure at the China site was similar, except that the sections were incubated with the Spotlight tissue pretreatment kit (Invitrogen, Carlsbad, CA, USA, cat #00-8401) and processed as previously described [27].

The FISH gene fusion assay had previously been validated in a pilot study, which confirmed a FISH assay defined *RET-KIF5B* positive NSCLC tumor by sequencing (Additional file 1). Assessment of FISH signal was carried out by investigators blinded to clinical response. Preliminary assessment was performed at 60x magnification to identify any alterations in the distribution of the red and green signals. When these were observed, 50 tumor cells were analyzed at 100x magnification for the number of red, green, paired red/green, gold, paired green/gold and blue signals in 10–15 tumor cells from up to four regions of the tumor section. A further region was then analyzed by a second blinded observer. To describe tumor heterogeneity, estimation of the proportion and distribution of cells with *RET* events was determined independently by two observers.

#### Immunohistochemistry

Sections were deparaffinized and rehydrated as described above. Antigen retrieval was performed at 110°C for 5 minutes in Target Retrieval Solution (pH 9.0; Dako, #S2367) in a RHS-2 microwave processor (Milestone, Sorisole, Italy) within a pressurized reaction vessel. Endogenous peroxidase activity was quenched by incubating the sections in 3% hydrogen peroxide for 20 minutes at room temperature and non-specific binding was blocked by incubating in serum-free protein block (Dako, #X0909) for 20 minutes at room temperature. Sections were labeled with a rabbit anti-RET monoclonal antibody (1:1000 dilution, clone EPR2871, Epitomics, Burlingame, CA, USA; see Additional file 1 for antibody specificity and immunohistochemistry validation; Additional file 1: Table S1) and RET expression was visualized with EnVision™ FLEX+ (Dako, #K8012). Sections were counterstained with hematoxylin. Cell lines (TT, MiaPaCa, SKNMC and Panc1) and tissue samples (human non-inflamed appendix and in-house NSCLC tissue microarray) were used as controls for RET immunostaining.

### Statistical analysis

For FISH analysis, tumors were categorized as positive for *RET* rearrangement if >10% of the tumor cells presented with broken-apart red and green *RET* signals; this could be further classified as positive for *RET-KIF5B* if the broken-apart red/green signal was accompanied by a paired green/gold signal. Tumors were categorized as low *RET* gene copy number gain if ≥40% of tumor cells had 4–6 copies of red/green *RET* signals. Tumors were categorized as amplified if >10% of tumor cells had ≥7 red/green signals or signal clusters. In the IHC analysis, tumor samples with >100 intact tumor cells were assessed by a system similar to that described previously [8]. Tumors were categorized as RET expression positive where the staining signal intensity was ++ or +++ (0 to +++ scale) in >10% of tumor cells. The objective response rate (ORR) was presented by RET biomarker status and treatment arm with corresponding 95% confidence intervals (CIs). Prevalence rates were estimated across all patients with a known result (including those not randomized to treatment) and were presented as a percentage with corresponding 95% CIs.

## Results

### Patients

From 4089 patients across the four NSCLC studies, 1291 and 1234 screened patients had tumor samples available for FISH and IHC analysis, respectively. Evaluable data were obtained for 944 (FISH; Additional file 1: Table S2) and 1102 (IHC; Additional file 1: Table S3) patients, with seven and eight patients donating FISH and IHC samples, respectively, not randomized to treatment. Failure rates in the IHC analysis (10.7%) were largely due to an inadequate number of tumor cells in the samples, whereas in the FISH analysis, failure rates (26.9%) were largely due to an inadequate number of tumor cells or sample quality. The median age of patients was 61 years; approximately two-thirds were white, with the remainder predominantly of Asian origin. Most patients (61%) presented with adenocarcinoma. Patient demographics and baseline characteristics for patients with tumor samples evaluable for FISH or IHC analysis and clinicopathologic characteristics of patients and their RET biomarker status are outlined in Additional file 1: Tables S2–S4, respectively.

### Prevalence of RET biomarkers

In this NSCLC patient population, the overall prevalence of *RET* rearrangements was 0.7% (95% CI 0.3–1.5) among patients with a known result. Seven tumor samples were classified as positive for *RET* rearrangements (vandetanib treatment, *n* = 3; comparator treatment, *n* = 4; Table 1; Figure 1a and b). Five of the seven *RET* rearrangements were *RET-KIF5B* and the other two had unknown fusion partners with *RET*. Single red or green signals without a corresponding 3′ or 5′ *RET* signal were occasionally seen in samples but were not scored as rearrangements.

**Table 1:** **Frequency of RET biomarkers in vandetanib Phase III NSCLC trial program**

Clinical study	RET biomarker, n (%) [95% CI]							
	*RET*rearrangement		*RET*amplification		Low*RET*gene copy number gain		RET expression	
	Van	Comp	Van	Comp	Van	Comp	Van	Comp
ZODIAC + ZEAL	1	2**	6	7	14	14	16	24
ZEPHYR	2	1	2	3	19	5	18	10
ZEST	0	1**	4	2	8	14	12	10
Untreated*	–		2		2		2	
Non-Asian	5/632 (0.8)		19/632 (3.0)		49/632 (7.7)		52/756 (6.9)	
	[0.3–1.8]		[1.8–4.6]		[5.7–10.0]		[5.2–8.9]	
Asian	2/305 (0.7)		7/305 (2.3)		27/305 (8.8)		40/346 (11.6)	
	[0.1–2.3]		[0.9–4.6]		[5.9–12.5]		[8.4–15.4]	
**Overall**	**7/937 (0.7)**		**26/937 (2.8)**		**76/937 (8.1)**		**92/1102 (8.3)**	
	**[0.3–1.5]**		**[1.8–4.0]**		**[6.4–10.0]**		**[6.8–10.1]**	

Comp, comparator; Van, vandetanib. *One patient randomized to ZODIAC and one randomized to ZEAL did not receive treatment. **One RET rearrangement with an unknown, non-*KIF5B* fusion partner was identified in the ZEAL comparator and one was identified in the ZEST comparator arm.

**Figure 2:**
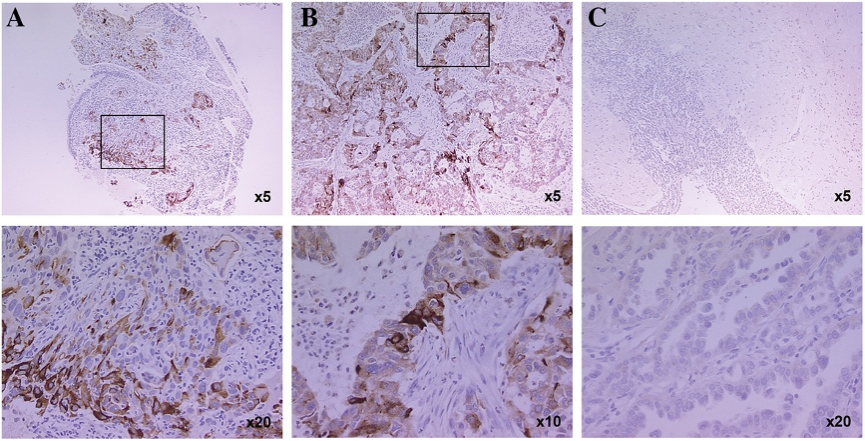
**Representative IHC images positive for RET expression. (A)** Tumor biopsies and **(B)** resections. **(C)** Negative (weak) staining.

*RET* gene amplifications and low *RET* gene copy number gains were reported in 26 (2.8%; 95% CI 1.8–4.0) and 76 (8.1%; 95% CI 6.4–10.0) patients, respectively (Table 1; Figure 1c and d). RET expression was positive in samples from 92 (8.3%; 95% CI 6.8–10.1) patients (Table 1). Tumor cell immunostaining was generally cytoplasmic and diffuse (Figure 2). The prevalence of *RET* rearrangement, RET protein expression, *RET* amplification or low *RET* gene copy number gain was similar for Asian and non-Asian populations (Table 1).

In our study, four out of seven tumors that were positive for *RET* rearrangement expressed RET when evaluated with IHC. Of the remaining three samples, one *RET*-*KIF5B* and two *RET*-other rearrangements did not express any detectable RET protein when evaluated with IHC (Additional file 1: Table S5). In all IHC-positive cases, RET was predominantly cytoplasmic and typically of moderate to weak intensity. In two cases, RET was only detected in the focal areas of tumor cells. Weak staining for RET was also observed in the stroma of three *RET*-rearrangement-positive tumors. Of the seven *RET*-rearrangement-positive tumors, only one showed amplification which was also positive when evaluated with IHC; this case also showed weak RET staining across all of the stroma present in the sample.

### Clinical outcome by positive RET biomarker status

None of the three vandetanib-treated *RET*-rearrangement-positive patients had an objective response (Table 2). Radiologic evidence of tumor shrinkage was observed in two of these patients (ZEPHYR, 23% and 33% shrinkage of target lesions). However, a response could not be confirmed at the next visit. One patient in ZODIAC who received docetaxel alone had a confirmed objective response and 32% shrinkage of target lesions at day 85 (Table 3). The ORRs were similar for the vandetanib and comparator arms for patients positive for *RET* amplification (8.3% vs 8.3%), those with low *RET* gene copy number gain (9.8% vs 9.1%) or those positive for RET protein expression (15.2% vs 13.6%, Table 2). In conclusion, we considered there were too few *RET*-rearrangement-positive patients to draw definitive conclusions regarding efficacy in this patient population. However, the lack of additional benefit observed in the higher prevalence biomarker-positive groups of *RET* amplification and low *RET* gene copy number gain is consistent with these biomarkers having little predictive utility to identify those patients who will benefit from vandetanib therapy.

**Table 2:** **ORRs (RECIST) in patients positive for RET biomarkers**

Clinical study	Objective responses, n/N (%)							
	*RET*rearrangement		*RET*amplification		Low*RET*gene copy number gain		RET expression	
	Van	Comp	Van	Comp	Van	Comp	Van	Comp
ZODIAC + ZEAL	0/1	1/2	1/6	1/7	2/14	2/14	5/16	3/24
ZEPHYR	0/2	0/1	0/2	0/3	0/19	0/5	0/18	0/10
ZEST	0/0	0/1	0/4	0/2	2/8	1/14	2/12	3/10
**Overall**	**0/3 (0.0)**	**1/4 (25.0)**	**1/12 (8.3)**	**1/12 (8.3)**	**4/41 (9.8)**	**3/33 (9.1)**	**7/46 (15.2)**	**6/44 (13.6)**

**Table 3:** **Clinicopathologic characteristics of seven patients positive for** ***RET*** **rearrangements**

Study	Age (years)	Sex	Race	Smoking status*	Histology	*EGFR*status	*KRAS*status	Dose/day	Exposure	RECIST response	Tumor shrinkage	Reason for discontinuation	RET partner	% cells with rearrangements detected
**Vandetanib**														
ZODIAC	68	F	Asian	Non-smoker	Adenocarcinoma	Mutation negative	Negative	100 mg	21 days	Progressive disease	–	Progressive disease	KIF5B	50%
ZEPHYR	69	F	White	Non-smoker	Adenocarcinoma	Mutation negative; amplification positive	Negative	300 mg	180 days	Stable disease	23% shrinkage of target lesions	Adverse event	KIF5B	75–100%
ZEPHYR	59	F	Asian	Non-smoker	Adenocarcinoma	Mutation negative; amplification positive	Negative	300 mg	57 days	Progressive disease	33% shrinkage of target lesions (progressive disease in non-target lesions)	Progressive disease	KIF5B	50–75%
**Comparator**														
ZODIAC	59	F	White	Ex-smoker	Adenocarcinoma	Mutation negative	Unknown	–	Six cycles docetaxel	Partial response (day 85); progressive disease (day 210)	32% shrinkage of target lesions at day 85	Completed six cycles	KIF5B	75–100%
ZEAL**	58	M	White	Ex-smoker	Large cell carcinoma	Mutation negative	Negative	–	Five cycles pemetrexed	Progressive disease (day 245)	None	Completed five cycles	Not known	75–100%
ZEPHYR	57	M	White	Ex-smoker	Adenocarcinoma	Mutation negative	Negative	–	26 days placebo	Progressive disease (day 25)	None	Progressive disease	KIF5B	75–100%
ZEST**	70	M	White	Ex-smoker	Adenocarcinoma	Mutation negative; amplification positive	Negative	–	315 days erlotinib	Progressive disease (day 166)	None	Progressive disease	Not known	25–50%

F, female; M, male. *Non-smoker = never smoked >20 g tobacco in lifetime; ex-smoker = stopped smoking ≥1 year ago; occasional smoker = <1 tobacco product per day; habitual smoker = ≥1 tobacco product per day. **RET rearrangements with unknown, non-*KIF5B* fusion partners.

## Discussion

In this retrospective study, the overall prevalence of *RET* rearrangements within the Phase III vandetanib NSCLC clinical program was determined as 0.7% among patients with a known *RET* rearrangement status. We found consistent frequencies of *RET* rearrangement in Asian (0.7%) and non-Asian patients (0.8%). In general, *RET* rearrangement prevalence rates may be considered as low and may vary according to the proportions of smokers, racial origin and histological subtype in the study population. Prevalence rates of *RET* rearrangement in Asian populations have been reported at 1–2% for NSCLC [5,11] and lung adenocarcinoma [2,3,7,12], and were estimated as high as approximately 6% in lung adenocarcinoma [4]. Our own study contains a high proportion of non-Asian patients (67%, Additional file 1: Table S2) and smokers/ex-smokers (77%, Additional file 1: Table S2), in contrast to previous reports on *RET* rearrangement prevalence rates [3,5,7,11,12], in which study populations were either entirely or largely Asian and non-smokers.

*RET* rearrangements have previously been reported in squamous cell carcinoma [5], lung neuroendocrine tumor [5] and adenosquamous tumor [11]; however, the majority occur in adenocarcinomas. This is consistent with our findings, which show a higher prevalence of *RET* rearrangements in patients with adenocarcinoma (1.2%, 6/510) compared with those in other histology types (0.2%, 1/427). Our data are not in agreement with a number of studies that report a higher frequency of *RET* rearrangements in non-smokers compared with smokers/ex-smokers; in our study, we have observed three and four *RET* rearrangements, respectively (Table 3) [2,5,11]. As in previous studies, we did not observe co-occurrence of *RET* rearrangements with *EGFR* and *KRAS* mutations. Interestingly, one of the three *RET-KIF5B* tumors reported by Go *et al.* [28] in lung adenocarcinomas negative for *KRAS* and *EGFR* mutations and *ALK* rearrangements was from a smoker. However, all of these observations should be interpreted with caution given the small numbers.

The techniques used to identify *RET* rearrangement genes in previously reported studies were sequencing [2-4] or reverse transcription-polymerase chain reaction followed by verification with FISH [11], sequencing [5,12] or differential expression of 3′ and 5′ *RET* exons [7,9,11]. Some of these techniques may underestimate the frequency of *RET* rearrangement genes by failing to detect fusions to partner genes other than *KIF5B*. We used a four-probe FISH assay to detect *RET* rearrangements. This technique is highly sensitive in detecting chromosomal rearrangements and has the advantage of detecting other partners or isoforms, though it is not known whether all these rearrangements are functional. For example, in a study using a split FISH assay to evaluate 1528 lung cancers, 22 tumors were detected with split *RET* signals, of which 12 were confirmed as fusions with *KIF5B* and one with *CCDC6* [10] and the remaining nine tumors showed little or no expression of the RET kinase domain.

Although the prevalence of *RET* rearrangements in NSCLC patients is low, RET inhibition may be efficacious within a subset of patients who carry these genetic aberrations. In this study, there were too few vandetanib-treated patients with *RET* rearrangements to form conclusions regarding association with efficacy. A number of studies have reported increased expression of RET protein in NSCLC tumor cells, not necessarily associated with *RET* rearrangements [2,3,8,11]. This led us to investigate both IHC and *RET* copy number gain as possible predictive biomarkers for vandetanib response. No difference was observed in the ORRs between vandetanib and comparator arms for IHC and copy number analyses.

In our study, we observed *RET-KIF5B* and other *RET* rearrangements in samples that were negative for RET protein expression. This observation is in line with previous studies of NSCLC samples which have used a range of anti-RET antibodies (including the Epitomics EPR2871 antibody we have used here) and differing IHC techniques [2,3,8,28]. Sasaki *et al.* [8] reported three cases of RET translocation (from 371 NSCLC samples), all of which had weak positive cytoplasmic staining when evaluated with a 3F8 anti-RET mouse monoclonal antibody (Vector Laboratories, Peterborough, UK). In contrast, when using the EPR2871 antibody used in our study, weak, moderate and strong staining were reported for the three RET translocation positive samples; this suggests that apparent RET expression is dependent on both the antibody and the local IHC protocol used. Another study has reported weak to strong RET expression with IHC when using the 3F8 anti-RET antibody; however, only one of the RET IHC positive cases was also *RET-KIF5B* positive [3]. Using the EPR2871 antibody, Kohno *et al.* reported 48/222 NSCLC cases to express RET in the absence of a *RET* fusion; all cases of *RET-KIF5B* were also RET positive with IHC [2]. Go *et al.* [28] used three different anti-RET antibodies to screen 53 NSCLC cases for RET protein expression. RET IHC positive cases were defined as those with >30% of cells expressing cytoplasmic RET. Three samples that were *RET*-fusion positive with whole transcript sequencing were negative for RET with IHC, whereas RET protein was identified in four samples, none of which harbored a *RET-KIF5B* rearrangement. Taken together, these NSCLC studies, along with our results, suggest that not all cases of *RET-KIF5B* or other *RET* rearrangements express RET protein when evaluated with IHC. RET protein appears to be largely cytoplasmic; however, considerable inter-patient variation and heterogeneity among tumor cells within individual tumors is observed.

Investigation of RET inhibitors in NSCLC patients with a documented confirmation of a *RET* rearrangement is an active area of research with three clinical studies currently ongoing (NCT01639508, NCT01823068 and NCT01813734). Results from a study on the use of vandetanib in *RET*-rearrangement-positive NSCLC patients (NCT01823068) should provide further insight into the role of vandetanib in this patient population. Preliminary data from NCT01639508, a prospective Phase II trial investigating the use of cabozantinib, a small-molecule inhibitor of MET, VEGFR2 and RET, has been published [6]. For the first three patients treated with cabozantinib, two patients showed confirmed partial clinical responses and the third patient had prolonged stable disease approaching 8 months [6]. A case study in a patient with poorly differentiated lung adenocarcinoma, positive for a *RET-KIF5B* and refractory for previous chemotherapy, is also noteworthy. In this patient, 4 weeks of treatment with vandetanib 300 mg once daily produced a fluorodeoxyglucose-positron emission tomography/computed tomography response [29]. In addition, in a preliminary study in which two heavily pretreated patients with confirmed *RET* rearrangements were treated with vandetanib, stable disease was observed following treatment [30].

## Conclusions

This study has demonstrated an overall prevalence of 0.7% for *RET* rearrangements in a large Phase III NSCLC patient population among patients with a known determination of *RET* rearrangement status composed predominantly of non-Asian patients and smokers. *RET* rearrangements were found most frequently, but not exclusively, in adenocarcinomas and occurred in tumors negative for other driver mutations, in agreement with previous reports in predominantly Asian populations. The prevalence of *RET* rearrangement is too low in this unselected population to determine whether *RET*-rearrangement-positive patients can be effectively treated with RET inhibitors, such as vandetanib. Changes in *RET* gene copy number and level of RET protein expression are more frequent aberrations than *RET* rearrangement in this NSCLC population, but also do not provide predictive markers for response to vandetanib. Results from additional studies, specifically in *RET*-rearrangement-positive NSCLC patients, are needed to determine whether this patient population can be effectively treated with RET inhibitors, such as vandetanib. If these studies support *RET* rearrangements as a clinically relevant target, then screening of NSCLC patients for *RET* rearrangements may become part of standard care.

## Additional file

Additional file 1:
**Supporting information for FISH assay validation, Western blotting/IHC antibody specificity, localization of RET in tumor tissue sections, patient demographics and baseline characteristics for patients with tumor samples evaluable for FISH and/or IHC analysis.**
