# Supplementary material for: A retrospective analysis of RET translocation, gene copy number gain and expression in NSCLC patients treated with vandetanib in four randomized Phase III studies
Source: BMC Cancer. 2015 Mar 23;15:171. doi: 10.1186/s12885-015-1146-8 (PMC4412099; doi:10.1186/s12885-015-1146-8)
Supplement: Additional file 1: — Supporting information for FISH assay validation, Western blotting/IHC antibody specificity, localization of RET in tumor tissue sections, patient demographics and baseline characteristics for patients with tumor samples evaluable for FISH and/or IHC analysis. [file 12885_2015_1146_MOESM1_ESM.docx]

**A retrospective analysis of *RET* translocation, gene copy number gain and expression in NSCLC patients treated with vandetanib in four randomized Phase III studies**

**Supporting information**

**FISH assay validation**

As part of the assay validation, the FISH assay was run on metaphase spreads to demonstrate the specificity of the probes (Fig S1A). To test sensitivity, the FISH assay was run on a panel of eight tumor cell lines, which had been characterized as negative for RET rearrangements. No false positive events were detected in the cell lines. A KIF5B-RET fusion was detected by the FISH assay in a sample from the pilot study, for this sample there was sufficient material to perform confirmatory analysis (Fig S1B, PCR primer sequences detailed below). RT-PCR and sequencing was performed on this subject, which confirmed the presence of a KIF5B-RET fusion (Fig S1C). Further analysis of study samples that were found to be positive for rearrangement of RET by FISH, was not possible as a result of insufficient remaining tissue.

*Primer sequences:*

Forward: 5’-AAAAACGAGCAGCTGAGATGATGGC-3’

Reverse: 5’-CGTTGCCTTGACCACTTTTCCAAAT-3’

**Figure S1. Verification of FISH assay specificity and sensitivity. (A) Metaphase spread showing specifity of the 4 FISH assay probes. (B) Lung FFPE sample with RET split and KIF5B-RET fusion detected by FISH. (C) RT-PCR plus sequencing confirming presence of KIF5B-RET fusion**

**
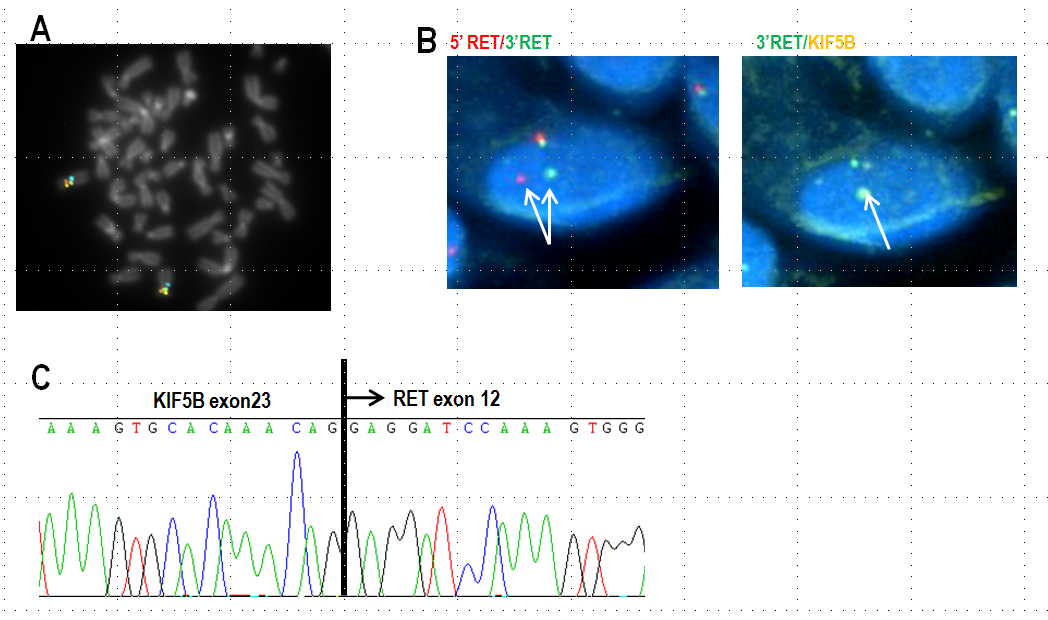
**

**Antibody specificity – Western blotting**

Cell lysates were prepared in RIPA buffer, separated by denaturing gel electrophoresis (4–12% pre-cast gels) and analyzed by Western blotting using the Epitomics anti-RET monoclonal antibody (1 in 10,000 dilution). A 150/170 kDa doublet was detected in lysates from MZ-CRC-1 and TT cells (medullary thyroid tumor cell lines positive for RET expression) consistent with non-glycosylated and glycosylated forms of RET [1]. The corresponding bands were absent in lysates prepared from HeLa cells (negative for RET expression). Molecular weight markers and size in kDa are indicated in Fig S2.

**Figure S2. RET Western blot analysis of MZ-CRC-1, TT and HeLa cells**

***
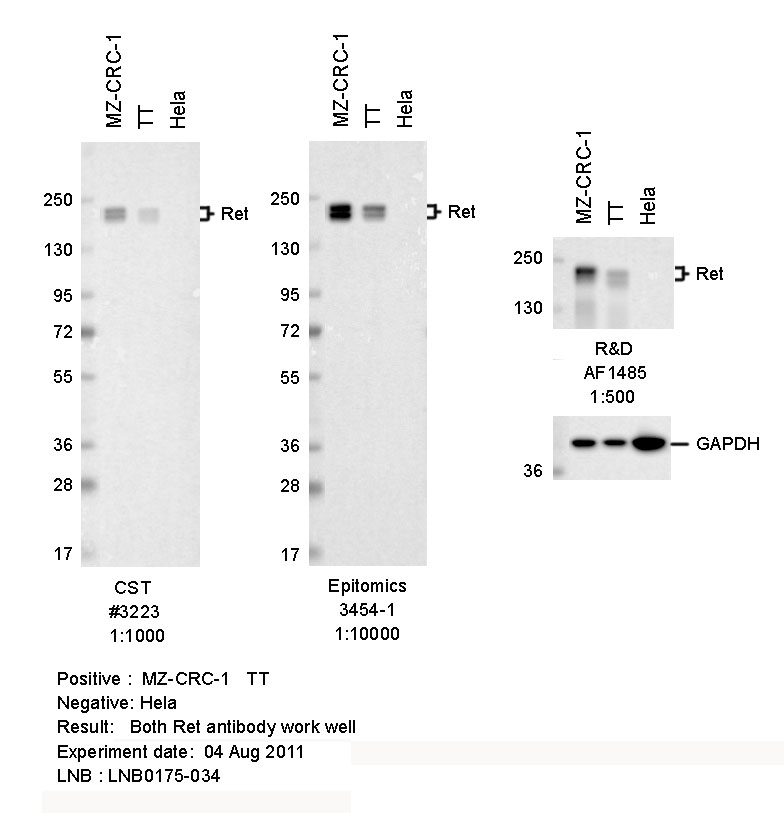
***

Alternative splicing of human RET results in the expression of three isoforms with distinct C-termini, known as RET9, RET43 and RET51. The Epitomics EPR2871 antibody is specific for the long (RET51) isoform of RET and does not detect the shorter (RET43 and RET9) isoforms. All three isoforms are co-expressed and RET9 and RET51 are the more abundant transcripts; the RET43 transcript has been reported to be expressed only at very low levels in all tissues examined [2], and RET51 may be a more potent transforming isoform [3].

Although the Epitomics antibody may underestimate the overall abundance of RET protein, it will detect the more abundant and transformationally relevant RET isoform, and the relative proportions of RET9 and RET51 are broadly similar, at least in a range of pheochromocytomas and MEN2 tumors [4]. Currently, there is no information to suggest that one or the other of RET9 or RET51 isoforms are more frequently associated with RET fusions in papillary thyroid or NSCLC cancers.

**Antibody specificity - Immunohistochemistry**

Examples of RET expression in are presented in Fig S3 (A) SK-N-MC neuroepithelioma cells (RET –ve) and (B) TT medullary thyroid cancer cells (RET +ve). Tumor cell pellets were fixed in formalin and processed into paraffin blocks for immunohistochemistry (IHC) using the Epitomics anti-RET monoclonal antibody.

**Figure S3. Immunohistochemical analysis of RET expression in A) SK-N-MC neuroepithelioma cells (RET –ve) and (B) TT medullary thyroid cancer cells (RET +ve)**

**Immunohistochemistry – NSCLC tissue samples**

IHC was carried out on 4-μM tissue sections following antigen retrieval (Dako S2367, pH 9.0, 110°C for 5 min) and using Epitomics anti-RET monoclonal antibody (Cat. 3454-1, 1:1000) and Dako Envision Flex+ detection system. Sections with >100 intact tumor cells were scored and staining intensity was assessed according to the following: – 0, weak; 1–2, weak/moderate; 3, strong staining in >10% of tumor cells. Tissue sections were batched for staining and each IHC run included the following controls to monitor assay performance; cell line pellets from; TT medullary thyroid tumor cells (strong positive, staining intensity 3+), MiaPaCa (weak positive, staining intensity 0–1+) and Panc1 (weak positive, staining intensity 1–2+) pancreatic tumor cells, SK-N-MC neuroepithelioma cells (negative, staining intensity 0), sections from tissue microarrays representing NSCLC (adenocarcinomas and squamous cell cancer, staining intensities 0–3), and human non-inflamed appendix (nerve and ganglia RET positive cells). Only samples with >100 intact tumor cells were scored.

Localization of RET staining in tumor tissue sections was recorded (Table S1); scoring was as follows: positive IP cytoplasm = predominant intensity (2 or 3) cytoplasm; positive Imax cytoplasm = maximum intensity (2 or 3) cytoplasm; positive IP membrane = predominant intensity (2 or 3) membrane; positive Imax membrane = maximum intensity (2 or 3) membrane. Percentages were rounded up or down to the nearest whole number, and 11% of samples were judged to not have scorable tumor for any reason (eg tissue quality, % tumor cell content). Using the criteria set for the study (positive = predominant intensity 2 or 3 in more than 10% of tumor cells) the overall positivity rate by IHC was 8% (range 2–12); if maximum intensity 2/3 was counted then the rate almost doubled to 15% (range 4–23).

Tumor cell staining was generally cytoplasmic and diffuse within cells although in a minority of cases there was coarse granular deposition. Membrane staining was present in a small number of cases associated with cytoplasmic staining (apart from one case) and was observed as complete, incomplete and/or luminal. Lumen positivity was a feature of some papillary pattern cancers. There was no evidence of clear nuclear localization and occasional nuclear positivity may have been a reflection of poor preservation or antigen over retrieval for the individual sample.

**Table S1. Localization of RET staining in tumor tissue sections**

| **Study** | **Total** | **No tumor,  n (%)** | **Tumor** | **Positive IP cytoplasm, n (%)** | **Positive Imax cytoplasm, n (%)** | **Positive Imax cytoplasm, n (%)** | **Positive Imax cytoplasm, (%)** |
| --- | --- | --- | --- | --- | --- | --- | --- |
| **ZODIAC** | 374 | 33 (9) | 341 | 39 (11) | 66 (19) | 4 (1) | 4 (1) |
| **ZEAL** | 133 | 21 (16) | 112 | 2 (2) | 5 (4) | 2 (2) | 2 (2) |
| **ZEPHYR** | 248 | 24 (10) | 224 | 27 (12) | 52 (23) | 1 (1) | 1 (1) |
| **ZEST** | 383 | 41 (11) | 342 | 21 (6) | 46 (13) | 5 (1) | 5 (1) |

**Table S2. Patient demographics and baseline characteristics for patients with tumor samples evaluable for FISH analysis**

|  | **Clinical trial** | | | | | | | |  |
| --- | --- | --- | --- | --- | --- | --- | --- | --- | --- |
|  | **ZODIAC** | | **ZEAL** | | **ZEPHYR** | | **ZEST** | | **Overall** |
|  | Vandetanib *n* = 153 | Comparator *n* = 166 | Vandetanib *n* = 46 | Comparator *n* = 47 | Vandetanib *n* = 151 | Comparator *n* = 59 | Vandetanib *n* = 158 | Comparator *n* = 157 | *N* = 937* |
| **Median age, years (range)** | 61  (28–80) | 59  (20–81) | 60  (30–82) | 62  (35–79) | 61  (36–84) | 62  (45–80) | 62  (37–82) | 62  (26–83) | 61  (20–84) |
| **Sex, male/female** | 109/44 | 116/50 | 29/17 | 28/19 | 81/70 | 27/32 | 91/67 | 102/55 | 583/354 |
| **Race, *n* (%)**  White  Black  Asian  Other | 101 (66)  0 (0)  49 (32)  3 (2) | 96 (58)  1 (1)  61 (37)  8 (5) | 38 (83)  3 (7)  4 (9)  1 (2) | 41 (87)  1 (2)  5 (11)  0 (0) | 73 (48)  1 (1)  74 (49)  3 (2) | 34 (58)  0 (0)  23 (39)  2 (3) | 113 (72)  1 (1)  43 (27)  1 (1) | 108 (69)  2 (1)  46 (29)  1 (1) | 604 (64)  9 (1)  305 (33)  19 (2) |
| **WHO PS, *n* (%)**  0  1  2  Unknown | 64 (42)  86 (56)  2 (1)  1 (1) | 63 (38)  101 (61)  0 (0)  2 (1) | 24 (52)  18 (39)  4 (9)  0 (0) | 17 (36)  29 (62)  1 (2)  0 (0) | 51 (34)  78 (52)  22 (15)  0 (0) | 13 (22)  34 (58)  11 (19)  1 (2) | 46 (29)  97 (61)  15 (9)  0 (0) | 51 (32)  92 (59)  13 (8)  1 (1) | 329 (35)  535 (57)  68 (7)  5 (<1) |
| **Smoking status^†^, *n* (%)**  Non-smoker  Ex-smoker  Occasional smoker  Habitual smoker | 22 (14)  75 (49)  1 (1)  55 (36) | 26 (16)  69 (42)  0 (0)  71 (43) | 9 (20)  24 (52)  1 (2)  12 (26) | 8 (17)  25 (53)  0 (0)  14 (30) | 68 (45)  73 (48)  1 (1)  9 (6) | 27 (46)  24 (41)  2 (3)  6 (10) | 25 (16)  88 (56)  1 (1)  44 (28) | 33 (21)  87 (55)  2 (1)  35 (22) | 218 (23)  465 (50)  8 (1)  246 (26) |
| **Histology, *n* (%)**  Adenocarcinoma  Adenocarcinoma: bronchioloalveolar  Adenosquamous carcinoma  Squamous cell carcinoma  Large cell carcinoma  Other | 80 (52)  8 (5)  1 (1)  49 (32)  8 (5)  7 (5) | 92 (55)  5 (3)  3 (2)  40 (24)  15 (9)  11 (7) | 23 (50)  2 (4)  0 (0)  15 (33)  3 (7)  3 (7) | 25 (53)  3 (6)  0 (0)  16 (34)  1 (2)  2 (4) | 95 (63)  18 (12)  4 (3)  25 (17)  3 (2)  6 (4) | 37 (63)  8 (14)  1 (2)  6 (10)  6 (10)  1 (2) | 82 (52)  4 (3)  1 (1)  44 (28)  14 (9)  13 (8) | 76 (48)  9 (6)  3 (2)  42 (27)  18 (11)  9 (6) | 510 (54)  57 (6)  13 (1)  237 (25)  68 (7)  52 (5) |
| **EGFR mutation status, *n* (%)**  Positive^‡^  G719X  Exon 19 deletion  S768I  Exon 20 insertion  L858R  L861Q  Negative  Unknown | 20 (13)  1 (1)  8 (5)  2 (1)  0 (0)  11 (7)  0 (0)  103 (67)  31 (20) | 12 (7)  0 (0)  8 (5)  0 (0)  0 (0)  4 (2)  0 (0)  116 (70)  38 (23) | 1 (2)  0 (0)  1 (2)  0 (0)  0 (0)  0 (0)  0 (0)  31 (67)  14 (30) | 3 (6)  0 (0)  1 (2)  0 (0)  0 (0)  2 (4)  0 (0)  31 (66)  13 (28) | 43 (28)  2 (1)  24 (16)  2 (1)  1 (1)  15 (10)  1 (1)  71 (66)  38 (25) | 11 (19)  1 (2)  9 (15)  0 (0)  1 (2)  1 (2)  0 (0)  33 (56)  15 (25) | 19 (12)  0 (0)  11 (7)  0 (0)  0 (0)  7 (4)  1 (1)  113 (72)  26 (16) | 22 (14)  1 (1)  9 (6)  1 (1)  0 (0)  10 (6)  2 (1)  109 (69)  26 (17) | 131 (14)  5 (1)  71 (8)  5 (1)  2 (<1)  50 (5)  4 (<1)  607 (65)  201 (21) |
| **EGFR amplification status, *n* (%)**  Positive  Negative  Unknown | 49 (32)  80 (52)  24 (16) | 45 (27)  88 (53)  33 (20) | 21 (46)  21 (46)  4 (9) | 15 (32)  30 (64)  2 (4) | 37 (24)  80 (53)  34 (23) | 19 (32)  31 (52)  9 (15) | 55 (35)  82 (52)  21 (15) | 32 (20)  93 (59)  32 (20) | 273 (29)  505 (54)  159 (17) |
| **KRAS status, *n* (%)**  Mutation positive  Mutation negative  Mutation unknown | 19 (12)  99 (65)  35 (23) | 20 (12)  104 (63)  42 (25) | 5 (11)  25 (54)  16 (35) | 8 (17)  22 (47)  17 (36) | 16 (11)  96 (64)  39 (26) | 9 (15)  36 (61)  14 (24) | 32 (20)  91 (58)  35 (22) | 22 (14)  107 (68)  28 (18) | 131 (14)  580 (62)  226 (24) |

WHO PS, World Health Organization performance status
*Eight patients with a known result were not randomized to treatment
^†^Non-smoker = never smoked >20 g tobacco in lifetime; ex-smoker = stopped smoking ≥1 year ago; occasional smoker = <1 tobacco product per day; habitual smoker = ≥1 tobacco products per day
^‡^Patients could harbor >1 EGFR mutation

**Table S3. Patient demographics and baseline characteristics for patients with tumor samples evaluable for IHC analysis**

|  | **Clinical trial** | | | | | | | |  |
| --- | --- | --- | --- | --- | --- | --- | --- | --- | --- |
|  | **ZODIAC** | | **ZEAL** | | **ZEPHYR** | | **ZEST** | | **Overall** |
|  | Vandetanib *n* = 183 | Comparator *n* = 186 | Vandetanib *n* = 51 | Comparator *n* = 59 | Vandetanib *n* = 169 | Comparator *n* = 77 | Vandetanib *n* = 189 | Comparator *n* = 180 | *N* = 1094* |
| **Median age, years (range)** | 62  (28–80) | 60  (20–82) | 60  (30–82) | 60  (35–79) | 62  (36–84) | 60  (28–80) | 62  (37–82) | 62.5  (35–83) | 62  (20–84) |
| **Sex, male/female** | 129/54 | 129/57 | 35/16 | 38/21 | 86/83 | 35/42 | 109/80 | 117/63 | 678/416 |
| **Race, n (%)**  White  Black  Asian  Other | 125 (68)  0 (0)  54 (30)  4 (2) | 116 (62)  1 (1)  60 (32)  9 (5) | 42 (82)  3 (6)  4 (8)  2 (4) | 51 (86)  0 (0)  8 (14)  0 (0) | 86 (51)  2 (1)  78 (46)  3 (2) | 39 (51)  0 (0)  35 (45)  3 (4) | 132 (70)  2 (1)  54 (29)  1 (1) | 126 (70)  2 (1)  51 (28)  1 (1) | 717 (66)  10 (1)  344 (31)  23 (2) |
| **WHO PS, n (%)**  0  1  2  Unknown | 78 (43)  102 (56)  2 (1)  1 (1) | 72 (39)  112 (60)  0 (0)  2 (1) | 26 (51)  22 (43)  3 (6)  0 (0) | 21 (36)  36 (61)  2 (3)  0 (0) | 52 (31)  97 (57)  20 (12)  0 (0) | 14 (18)  47 (61)  15 (20)  1 (1) | 52 (28)  119 (63)  18 (10)  0 (0) | 61 (34)  100 (56)  18 (10)  1 (1) | 376 (34)  635 (58)  78 (7)  5 (<1) |
| **Smoking status^†^, n (%)**  Non-smoker  Ex-smoker  Occasional smoker  Habitual smoker | 27 (15)  91 (50)  1 (1)  64 (35) | 31 (17)  83 (45)  1 (1)  71 (38) | 9 (18)  30 (59)  1 (2)  11 (22) | 9 (15)  32 (54)  0 (0)  18 (31) | 83 (49)  75 (44)  3 (2)  8 (5) | 37 (48)  29 (38)  3 (4)  8 (10) | 27 (14)  111 (59)  1 (1)  50 (27) | 34 (19)  146 (81)  3 (2)  36 (20) | 257 (23)  597 (55)  13 (1)  266 (24) |
| **Histology, n (%)**  Adenocarcinoma  Adenocarcinoma: bronchioloalveolar  Adenosquamous carcinoma  Squamous cell carcinoma  Large cell carcinoma  Other | 100 (55)  8 (4)  2 (1)  55 (30)  9 (5)  9 (5) | 99 (53)  5 (3)  3 (2)  45 (24)  18 (10)  16 (9) | 27 (53)  2 (4)  0 (0)  17 (33)  3 (6)  2 (4) | 32 (54)  3 (5)  0 (0)  18 (31)  3 (5)  3 (5) | 108 (64)  24 (14)  3 (2)  26 (15)  2 (1)  6 (4) | 50 (65)  10 (13)  2 (3)  5 (7)  8 (10)  2 (3) | 100 (53)  5 (3)  2 (1)  50 (27)  16 (9)  16 (9) | 91 (51)  10 (6)  3 (2)  49 (27)  18 (10)  9 (5) | 607 (55)  67 (6)  15 (1)  265 (24)  77 (7)  63 (6) |
| **EGFR mutation status, n (%)**  Positive^‡^  G719X  Exon 19 deletion  S768I  Exon 20 insertion  L858R  L861Q  Negative  Unknown | 28 (16)  1 (1)  14 (8)  1 (1)  1 (1)  12 (7)  0 (0)  118 (65)  38 (21) | 13 (7)  0 (0)  8 (4)  0 (0)  0 (0)  5 (3)  0 (0)  129 (69)  44 (24) | 1 (2)  0 (0)  1 (2)  0 (0)  0 (0)  0 (0)  0 (0)  31 (61)  19 (37) | 4 (6)  0 (0)  1 (2)  0 (0)  0 (0)  3 (5)  0 (0)  38 (64)  17 (29) | 47 (28)  2 (1)  26 (15)  2 (1)  1 (1)  17 (10)  1 (1)  78 (46)  46 (27) | 16 (21)  1 (1)  12 (16)  0 (0)  1 (1)  1 (4)  0 (0)  35 (46)  26 (34) | 20 (11)  0 (0)  11 (6)  0 (0)  0 (0)  8 (4)  1 (1)  127 (67)  42 (22) | 25 (14)  1 (1)  11 (6)  1 (1)  0 (0)  11 (6)  2 (1)  121 (67)  34 (19) | 154 (14)  5 (<1)  84 (8)  4 (<1)  3 (<1)  57 (5)  4 (<1)  677 (62)  266 (24) |
| **EGFR amplification status, n (%)**  Positive  Negative  Unknown | 56 (31)  89 (49)  38 (21) | 44 (24)  95 (51)  47 (25) | 21 (41)  21 (41)  9 (18) | 17 (29)  33 (56)  9 (15) | 43 (25)  84 (50)  42 (25) | 23 (30)  30 (39)  24 (31) | 54 (29)  89 (47)  46 (24) | 38 (21)  97 (54)  45 (25) | 296 (27)  538 (49)  260 (24) |
| **KRAS status, n (%)**  Mutation positive  Mutation negative  Mutation unknown | 22 (12)  114 (62)  47 (26) | 19 (10)  115 (62)  52 (28) | 5 (10)  27 (53)  19 (37) | 8 (14)  29 (49)  22 (37) | 20 (12)  105 (62)  44 (26) | 10 (13)  42 (55)  25 (32) | 34 (18)  103 (55)  52 (28) | 23 (13)  115 (64)  42 (23) | 141 (13)  650 (59)  303 (28) |

WHO PS, World Health Organization performance status
*Eight patients with a known result were not randomized to treatment
^†^Non-smoker = never smoked >20 g tobacco in lifetime; ex-smoker = stopped smoking ≥1 year ago; occasional smoker = <1 tobacco product per day; habitual smoker = ≥1 tobacco products per day
^‡^Patients could harbor >1 EGFR mutation

**Table S4. Clinicopathologic characteristics of patients and their RET biomarker status**

|  | **RET fusion** | | | **RET amplification** | | | **RET expression** | | | **RET low copy number gain** | | |
| --- | --- | --- | --- | --- | --- | --- | --- | --- | --- | --- | --- | --- |
|  | **Positive** | **Negative** | **Unknown** | **Positive** | **Negative** | **Unknown** | **Positive** | **Negative** | **Unknown** | **Positive** | **Negative** | **Unknown** |
| **Age, mean (SD)** | 62.9 (5.81) | 60.6 (10.20) | 59.2 (10.68) | 58.2 (7.55) | 60.7 (10.23) | 59.2 (10.68) | 59.6 (9.37) | 60.9 (10.13) | 59.1 (10.73) | 58.0 (9.63) | 60.9 (10.19) | 59.2 (10.68) |
| **Males, %** | 43 | 62 | 61 | 79 | 62 | 61 | 51 | 63 | 61 | 66 | 62 | 61 |
| **Caucasian, %** | 71 | 64 | 57 | 67 | 64 | 57 | 53 | 67 | 56 | 61 | 65 | 57 |
| **Asian, %** | 29 | 33 | 38 | 29 | 33 | 38 | 44 | 30 | 39 | 36 | 32 | 38 |
| **EGFR mutation positive, %** | 0 | 14 | 2 | 17 | 14 | 2 | 22 | 13 | 1 | 18 | 14 | 2 |
| **KRAS positive, %** | 0 | 14 | 1 | 17 | 14 | 1 | 16 | 13 | >1 | 12 | 14 | 1 |
| **Adenocarcinoma, %** | 86 | 60 | 57 | 69 | 60 | 57 | 74 | 60 | 57 | 68 | 60 | 57 |
| **Squamous cell carcinoma, %** | 0 | 26 | 17 | 12 | 26 | 17 | 10 | 26 | 17 | 14 | 26 | 17 |
| **Smokers, %** | 57 | 77 | 60 | 81 | 76 | 60 | 62 | 78 | 60 | 74 | 77 | 60 |

**Table S5. RET-rearrangement, amplification, copy status and immunohistochemical analyses**

| **RET-rearrangement** | **RET-IHC** | **RET-amplification** | **RET-low copy** | **IHC staining intensity;  % of tumor RET-positive** | **IHC staining intensity;  % of stroma RET-positive** |
| --- | --- | --- | --- | --- | --- |
| RET-KIF5B | Negative | Negative | Negative | No staining | No staining |
| RET-KIF5B | Positive | Negative | Positive | Moderate; >50% | No stroma present |
| RET-other | Negative | Negative | Negative | No staining | Weak/focal; <10% |
| RET-KIF5B | Negative | Negative | Negative | Weak; >50% | Weak/focal; <10% |
| RET-KIF5B | Positive | Negative | Negative | Moderate/patchy; 10–50%. | No stroma present |
| RET-KIF5B | Positive | Positive | Negative | Strong/focal; <10% | Weak; 100% |
| RET-other | Negative | Negative | Positive | Weak/focal; <10% | No staining |

IHC, immunohistochemistry; sections with >100 intact tumor cells were scored and staining intensity was assessed according to the following: – 0, weak; 1–2, weak/moderate; 3, strong staining in >10% of tumor cells.

**References**

1. Takahashi M, Asai N, Iwashita T, Isomura T, Miyazaki K, Matsuyama M: **Characterization of the ret proto-oncogene products expressed in mouse L cells.** *Oncogene* 1993, **8**:2925-2929.

2. Carter MT, Yome JL, Marcil MN, Martin CA, Vanhorne JB, Mulligan LM: **Conservation of RET proto-oncogene splicing variants and implications for RET isoform function.** *Cytogenet Cell Genet* 2001, **95**:169-176.

3. Degl'innocenti D, Arighi E, Popsueva A, Sangregorio R, Alberti L, Rizzetti MG, Ferrario C, Sariola H, Pierotti MA, Borrello MG: **Differential requirement of Tyr1062 multidocking site by RET isoforms to promote neural cell scattering and epithelial cell branching.** *Oncogene* 2004, **23**:7297-7309.

4. Le HH, Charlet-Berguerand N, Gimenez-Roqueplo A, Mannelli M, Plouin P, de F, V, Thermes C: **Relative expression of the RET9 and RET51 isoforms in human pheochromocytomas.** *Oncology* 2000, **58**:311-318.
